# Pyrexia and Hyperpyrexia

**Published:** 1898-06

**Authors:** 


For the Texas Medical Journal.

### Pyrexia and Hyperpyrexia.

A remedy, to be useful to the physician in combating fever, must have the power to hasten the expulsion of dissolved carbonic acid; must aid the excretion of the abundant nitrogenized matters which load the urine—augmented always during pyrexia; must quiet and control violent metabolism, thus, in great degree diminishing the rapid combustion of the complex chemical molecules, thereby checking structural disintegration prior to hopeless dissolution; must cleanse, so to speak, the pathological fields where heat in excess is generated, rather than merely produce its hasty liberation. It is desirable that these favorable changes be brought about without, in the least degree, weakening the patient's energy, otherwise, the reduction of temperature costs the patient dearly. Shock and depletion are to be condemned—depletion being here used in its full meaning. The cold bath is a good measure, often indispensible, but the patient should be put into water of 98° F., the water thereafter cooled suddenly with ice until 60° or less, if necessary, be obtained. Measures that bleed a patient into his own veins by contraction of the arterioles are not desirable. There are forms of fever in which the tendency to over-production of heat remains unmolested even through the most marked diaphoresis and diuresis. After continuous and enormous sweating in acute rheumatism the temperature still crawls a little higher as time wears by. So, too, in septic fever. Here the phenomena of fever first mentioned, plus the action of ferments and altered organic matters in the blood are witnessed. Is not some neutralizing agent here needed; some remedy to reach and counteract the poison disturbing cell life; some remedy to clean the blood of poisonous floating detritus by the process known as asepsis? Is this not the most reliable way of taking the pressure and friction off of the capillary arterioles? Do we by using aconite, thereby contracting them, give permanent relief? No. Must we not endeavor to normalize the affinities of the blood and tissues by purifying the several cells of each, and rendering them permanently wholesome and immune by curative as well as palliative measures? Cold water relieves, but often other measures are alike necessary to a permanent cure. If septic matter has entered the column of blood cold water alone will not cure the incident fever. No matter how good a state of nerve-tension the patient may have, it will soon fail through the waverings of an enfeebled nutritive process; and now just what it should do, i. e., restrain hyperoxidation—it fails to do, therefore, the normal nerve-tension being gone, hyperpyrexia is engendered.

Wherefore it is seen that the first step is to disinfect the poisoned molecule, otherwise all measures else are but palliative and tentative, and at best offer incomplete service in the thermic battle. The most useful remedy in those fevers that do not cool with perspiration, namely, acute rheumatism and septic pyrexia, in which there is a "something" in the blood that certainly is a potent factor in the extension of the febrile process, is one which acts as an antiseptic and antipyretic. Viskolein is entirely the most carefully prepared remedy of this character yet come to our attention. Its action on the heart, if any at all, is certainly nugatory. In our hands it has rather seemed to act first through the nervous system, fostering a salutary tension, and furnishing in some cases the greatest degree of beatitude. The tranquilizing influence of ten minims of the sterile solution hypodermically administered to one of our pyæmic cases persisted in some ten hours, might easily be compared to the effect of Magendie's solution. Whether it acts as a solvent in the septic state has not yet been sufficiently determined, but it is quite likely; we believe from clinical observation that it does. And it is certain that it very materially promotes the removal of the increased products of oxidation by transpiration and excretion. The preparation when used hypodermically is at once put in contact with the foe, and the engagement takes place instantly. If, as seems probable, it acts as a solvent of altered organic excresences in the blood, how much better to introduce it immediately into the general column, since in this manner it also more readily reaches and calms the brain. J. M. D.

